# The impact of intranasal fluticasone on patients with obstructive sleep apnea: a prospective study

**DOI:** 10.1016/j.bjorl.2019.07.007

**Published:** 2019-08-31

**Authors:** Yuan-Yun Tam, I-Hung Shao, Chia-Chen Wu, Ming-Li Hsieh

**Affiliations:** a Taoyuan General Hospital, Department of Otorhinolaryngology – Head and Neck Surgery, Ministry of Health and Welfare, Taoyuan City, Taiwan; b Chang Gung University, Linkou Chang Gung Memorial Hospital, Department of Surgery, Division of Urology, Taoyuan City, Taiwan; c Chang Gung University, Linkou Chang Gung Memorial Hospital, Department of Otorhinolaryngology – Head and Neck Surgery, Taoyuan City, Taiwan

**Keywords:** Obstructive sleep apnea, Intranasal corticosteroid, Sleep quality, Daytime dysfunction

## Abstract

**Introduction:**

Obstructive sleep apnea is the most common type of sleep apnea, which is caused by complete or partial obstructions of the upper airway. Nasal obstruction is also considered as one of the independent risk factors of obstructive sleep apnea.

**Objective:**

Patients with obstructive sleep apnea.

**Methods:**

We enrolled patients with obstructive sleep apnea from June to December 2015 and treated them with intranasal corticosteroid spray for four weeks. Several parameters were obtained before and after the treatment, including Nasal Obstruction Symptom Evaluation scores, Pittsburgh Sleep Quality Index questionnaire and Epworth Sleepiness Scale questionnaire.

**Results:**

Fifty patients completed questionnaires prior to and following the intranasal fluticasone treatments. The average age was 39.7 ± 15.6 y, with a male to female ratio of 3:2. The post-treatment Epworth Sleepiness Scale, Pittsburgh Sleep Quality Index, and Nasal Obstruction Symptom Evaluation scores all indicated a decrease compared to pre-treatment scores, from 10.4 to 8.74, 7.86 to 6.66 and 9.08 to 6.48, respectively. A significant decrease was observed in the Nasal Obstruction Symptom Evaluation ≥10 group in all three categories, but not in the Nasal Obstruction Symptom Evaluation <10 group.

**Conclusions:**

Intranasal fluticasone treatment may be useful for patients with nasal obstruction-related obstructive sleep apnea to improve sleep quality and limit daytime dysfunction.

## Introduction

Obstructive sleep apnea (OSA) is the most common type of sleep apnea and is caused by complete or partial obstruction of the upper airway. Approximately 2–32.8% of adults are believed to have OSA, and it is most commonly diagnosed in middle-aged males.^1^, ^2^, ^3^ The prevalence rate varies according to different populations. OSA is characterized by repetitive episodes of shallow or paused breathing during sleep, despite effort to breathe. A reduction in blood oxygen saturation during sleep is typically observed in patients with OSA.

OSA is typically related to the elderly, decreased muscle tone, increased soft tissue around the airway (sometimes owing to obesity) and obstructive structural features.^2^ Patients may have more than one of these conditions. Nasal obstruction is also considered an independent risk factor for OSA.^3^ Patients with nasal diseases such as allergic rhinitis may exhibit increased Nasal Airflow Resistance (NAR), which can contribute to the development of upper airway obstruction during sleep.^4^

Several studies have reported a relationship between snoring and OSA in patients with nasal obstructions with various diseases.^3^, ^5^, ^6^ However, whether the treatment of nasal obstruction can improve the outcome of patients with OSA remains unclear.

Standard treatment modalities for OSA include life-style modifications such as avoiding alcohol and smoking,^1^ weight loss for overweight patients, avoiding medication that relaxes the central nervous system, Continuous Positive Airway Pressure (CPAP), and the use of mandibular advancement devices.^7^, ^8^ To date, there is not much evidence to support the use of medication or surgery,^7^, ^9^ especially from the subjective aspect.

Because nasal obstruction can substantially affect OSA, treatments for nasal obstructions should benefit some of the OSA patients. In this study, we used the Nasal Obstruction Symptom Evaluation (NOSE) scale to compare treatment outcomes of using intranasal fluticasone in patients with different nasal obstructive severity.

## Methods

### Participants

The ethics committee approved this prospectively designed study (CGH-LP104003). Between June and December 2015, patients that visited our otolaryngology department who had snoring problems and were suspected of having OSA were asked to participate in a Polysomnography (PSG) study before any medication was prescribed. Once OSA was confirmed, as defined by a respiratory disturbance index ≥5, patients were recruited into this study for evaluation. Informed consent was provided by the participants. Patients were excluded from the study if they refused the medical treatment or refused to participate in the study. Patients who had received intranasal steroid treatment or any nasal or oral surgeries were excluded.

### Data collection and study protocol

We conducted face-to-face surveys on the basis of three internationally validated, reliable, and widely used questionnaires, namely the NOSE scale,^10^ the Pittsburgh Sleep Quality Index (PSQI) questionnaire^11^, ^12^ and the Epworth Sleepiness Scale (ESS) questionnaire.^13^, ^14^ After their written informed consent was obtained, patients were instructed to complete the questionnaires during their first visit. The only medication prescribed was intranasal fluticasone (27.5 mcg of fluticasone furoate per spray) which was sprayed once daily in each nostril (total daily dose, 110 mcg). The duration of each interview was approximately 10–20 min. After four weeks of intranasal corticosteroid treatment, the PSQI and ESS questionnaires were completed again when the patients returned to the clinic.

### Measuring tools

The NOSE^10^ survey is a validated disease-specific instrument to measure nasal obstruction. It is widely used in otolaryngology practices to provide an objective measure of nasal obstruction.

The PSQI was used to evaluate sleep quality and disturbance over the previous month. It consisted of two parts. One part contained 19 items referring to subjective sleep quality. The first four were open questions and the 5th to the 19th items were rated on a 4 point scale. The item scores yielded seven subscores ranging from 0 to 3: sleep quality, sleep latency, sleep duration, sleep efficiency, sleep disturbance, sleep medication use, and daytime dysfunction caused by sleepiness. The total scores, ranging from 0 to 21, were obtained by adding the seven subscores. Numerous studies have indicated that a total PSQI score ≤5 indicates good sleep quality, whereas a total PSQI score >5 indicates poor sleep quality.^11^, ^15^, ^16^ The other part contained five items regarding objective sleep quality. These five items were rated on a 4 point scale and added together for a total score from 0 to 15.

The ESS consists of 8 questions that are rated on a 4 point scale ranging from 0 to 3. A total score between 0 and 24 was used to evaluate participants’ general level of daytime sleepiness and their average sleep propensity over the previous month. An ESS score >10 indicated significant sleepiness.^15^, ^16^

The participants underwent in-laboratory diagnostic Polysomnography (PSG) in our hospital. All included subjects had transcutaneous pulse oximetry. Respiratory airflow was measured by an external thermistor, CO_2_ monitor, and nasal pressure cannula. Respiratory effort was recorded using respiratory inductance plethysmography. The Apnea-Hypopnea Index (AHI) represents the number of apneas and hypopneas per hour of sleep. Apnea was defined as a decrease of 90% or greater from the previous baseline airflow as measured by an oronasal thermistor for at least 10 s. Hypopnea was defined as a partial obstructive event with diminution of air flow by more than 30% from baseline for at least 10 s as measured using a nasal pressure cannula. The arousal events were recorded as Arousal Index (AI), which represent the number of arousal invents per hour.

### Statistical analysis

All questionnaire results were analyzed with a paired-sample *t*-test to ascertain the changes caused by intranasal corticosteroid use over four weeks. We also analyzed the premedication and post medication parameters in groups that were divided according to NOSE scores (≥10 and <10). A univariate analysis was performed with a Pearson correlation to compare the relationship between all parameters. General parameters were as follows: gender, age, height, weight, body mass index, nocturia frequency and neck circumference. PSG parameters included the AHI and the AI. Questionnaire parameters included the aforementioned evaluation methods: ESS, NOSE, and PSQI. Patient outcome parameters assessed the following symptoms: nocturia, sleep quality, daytime dysfunction and nasal obstruction. A multivariate analysis for the same parameters was then performed with linear regression. All the statistical analyses were performed on a personal computer with the statistical package SPSS for Windows (Version 17.0, SPSS).

## Results

Fifty patients completed questionnaires prior to and following the intranasal fluticasone treatments. The average age was 39.7 ± 15.6 y, with a male to female ratio of 3:2. Patient characteristics are displayed in Table 1. All patients completed PSG, which revealed a mean AHI of 27.9 and AI of 138.2, respectively. The post-treatment ESS, PSQI and NOSE scores all indicated a decrease comparing to pre-treatment scores, from 10.4 to 8.74 (*p* <  0.001), 7.86 to 6.66 (*p* <  0.001) and 9.08 to 6.48 (*p* <  0.001), respectively.

**Table 1:** Demographic and clinical data of patients with sleep-disorders related to breathing who were treated with an intranasal corticosteroid spray.

	Mean	Range
Basic parameters (Male to Female = 38:12)		
Mean patient age (y)	39.7 ± 15.6	13‒85
Body height (cm)	166.1 ± 7.53	150‒180
Body weight (kg)	75.4 ± 16.9	45‒115
Body mass index (BMI) (kg/m^2^)	27.2 ± 5.15	18.4‒38.8
Neck circumference (cm)	38.1 ± 4.1	28‒46
PSG parameters		
AHI	27.85 ± 28.0	5.0‒87.7
Arousal index	138.2 ± 108.4	19‒457
Pre-treatment Questionnaire results		
ESS score	10.4 ± 6.0	1‒24
PSQI total score	7.86 ± 4.48	1‒21
NOSE score	9.08 ± 4.9	2‒20
Post-treatment Questionnaire results		
ESS score	8.74 ± 5.7	1‒24
PSQI total score	6.66 ± 0.5	1‒21
NOSE score	6.48 ± 0.6	2‒20

AHI, Apnea-Hypopnea Index.

A Pearson’s correlation was then performed to determine the predictors for the improvement of sleep quality (PSQI score), daytime dysfunction (ESS score), and nasal obstruction score (NOSE score); results are illustrated in Table 2. Following the univariate analysis, a multivariate analysis was performed using linear regression; the results are illustrated in Table 3.

**Table 2:** Univariate analysis of factors affecting sleep quality improvement, daytime dysfunction and nasal obstruction using Pearson’s correlation coefficient.

	Age	BH	BW	BMI	NC	AHI	AI	ESS	NOSE	PSQI	Sex
PSQI score change	−0.128	−0.321^a^	−0.349^a^	−0.267	−0.318^a^	−0.335^a^	−0.366^b^	0.397^b^	0.499^b^	0.668^b^	−0.327^a^
ESS score change	0.206	0.043	0.226	0.239	0.300^a^	0.140	0.150	0.359^a^	0.332^a^	0.013	−0.027
NOSE score change	0.197	0.022	0.138	0.156	0.268	0.190	0.350^a^	0.206	0.478^b^	−0.027	0.042

NC, Neck Circumference; AI, Arousal index.

a Correlation is significant at the 0.05 level (2 tailed).

b Correlation is significant at the 0.01 level (2 tailed).

**Table 3:** Multivariate analysis for factors affecting sleep quality improvement, daytime dysfunction and nasal obstruction using standardized coefficients.

	Age	BH	BW	BMI	AHI	AI	PSQI	ESS	NOSE
PSQI score change	−0.313^a^	0.951	−3.441	2.973	0.029	−0.333^a^	×	0.075	0.596^a^
ESS score change	0.164	0.166	−0.253	0.398	−0.051	−0.072	−0.177	×	0.392^a^
NOSE score change	0.215	0.889	−1.840	1.377	−0.160	0.528^a^	−0.014	0.312	×

AI, Arousal index.

a Correlation is significant at the 0.05 level.

Factors that correlated with sleep quality improvement in the univariate analysis were sex, Body Height (BH), Body Weight (BW), Neck Circumference (NC), AHI, AI, initial ESS score, and initial NOSE score (Table 2). However, only age, AI and initial NOSE score exhibited significance in the multivariate analysis (Table 3). Higher initial NOSE scores correlated to greater sleep quality improvement, whereas older age and a higher initial AI on PSG negatively affected sleep quality improvement. Neck circumference, initial ESS score, and initial NOSE score significantly correlated with daytime dysfunction improvement in the univariate analysis (Table 2), but the only significant predictor in the multivariate analysis for daytime dysfunction improvement was the initial NOSE score (Table 3). In both the univariate and multivariate analyses, nasal obstruction symptom improvement only exhibited a significant correlation with initial AI (Table 2, Table 3).

**Figure 1:**
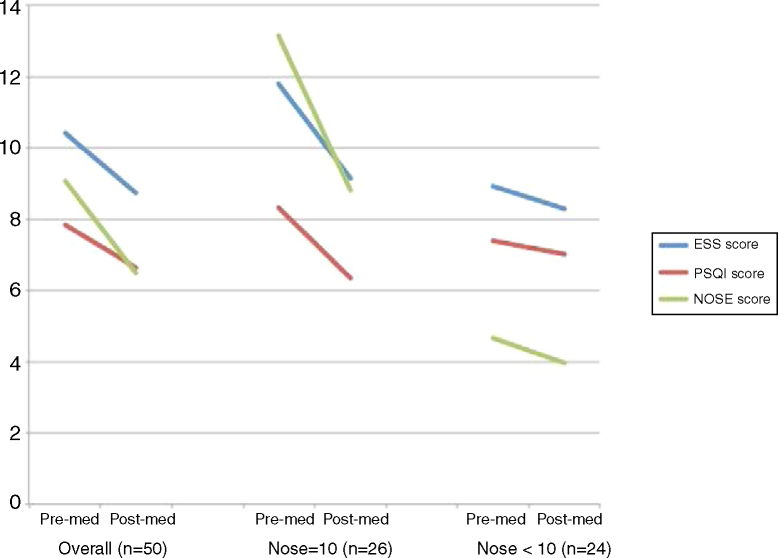
Improvement of clinical symptoms after prescription of intranasal steroid spray over the course of 4 weeks.

A paired-sample *t*-test was then performed to compare the ESS, PSQI, and NOSE score differences before and after intranasal fluticasone treatment over 4 weeks. The comparison was performed in an overall group and in divided groups, divided on the basis of initial NOSE scores (≥10 and <10) (Fig. 1). The divided groups comprised 26 and 24 patients, respectively. In the overall group, the ESS, PSQI, and NOSE scores decreased significantly after 4 weeks of intranasal fluticasone treatment from 10.42 to 8.74, 7.86 to 6.66 and 9.08 to 6.48, respectively. A significant decrease was observed in the NOSE ≥ 10 group in all three categories, but not in the NOSE < 10 group.

## Discussion

Although there is still insufficient evidence for medication such as intranasal steroids to be generally prescribed to patients with OSA, studies have previously examined the effect of intranasal steroids on specific groups of OSA patients. Upon reviewing previous studies, Kiely et al. conducted a randomized, placebo-controlled trial to evaluate the effect of intranasal steroids on patients with OSA and coexisting rhinitis, in which intranasal fluticasone improved AHI and nasal airway resistance.^17^ Brouillette et al. found that a 6 week nasal fluticasone treatment could decrease the frequency of mixed and obstructive apneas and hypopneas in pediatric OSA patients.^18^ An meta-analysis of randomized controlled trial had shown the objective improvement of intranasal corticosteroid on patients with OSA.^9^

In our study, we hypothesized that because nasal obstruction-related airway narrowing may lead to OSA in certain patients, intranasal steroids might relieve symptoms in patients with severe nasal obstruction problems. We used NOSE questionnaires to clarify the severity of nasal obstruction symptoms and divided the patients into two groups on the basis of NOSE scores greater than 10 and equal to or less than 10. A higher NOSE score was the only independent factor that was significantly relevant to sleep quality and daytime dysfunction improvement. After we divided patients according to NOSE scores, significant improvements in the three symptomatic scores (ESS, PSQI, and NOSE) appeared only in the group of NOSE scores ≥10. Therefore, intranasal steroid treatment may benefit patients with severe nasal obstructions complaining subjective reported symptoms such as sleep quality and daytime dysfunction, but the effect was minimal on patients without obvious nasal obstruction problems. In another study regarding nasal surgery on OSA patients with chronic nasal obstruction, Li et al. reported that nasal surgery resulted in an effective reduction of daytime sleepiness and snoring; however, the efficacy in treating OSA was limited.^19^ Similar results from future studies might further establish that treatment for nasal obstruction could benefit selected patients.

As previously mentioned, nasal obstruction can lead to increased nasal airway resistance, which contributes to upper airway obstruction during sleep and is a risk factor for OSA.^3^, ^4^ This study employed NOSE scores as a screening tool to determine which OSA patients’ condition likely resulted from a nasal obstruction and determined that intranasal steroid treatment may be an effective conservative treatment.

Aside from treatment selection applications, it has been suggested that NOSE could serve as a simple screening instrument instead of ESS for patients at risk of undiagnosed OSA and special perioperative needs.^20^

Current treatments for nasal obstruction such as intranasal steroids or nasal surgery have yet to be established as viable OSA treatment methods.^7^ However, for clinical cases wherein patients cannot modify their lifestyles or tolerate CPAP therapy and mandibular advancement devices, physicians may consider intranasal steroids, particularly because alternative options are limited. However, more evidence is required to support the viability of this option. Furthermore, NOSE scores could potentially be used as a screening scale for early detection of at-risk patients.

The limitations of this study were its small sample size and its lack of PSG follow-up after the 4 week treatment period. If more objective surveys such as rhinomanometry could be conducted, the effects and mechanisms of intranasal steroid treatment might become clearer.

## Conclusion

Intranasal fluticasone treatment may be useful for patients with nasal-obstruction-related OSA to improve sleep quality and limit daytime dysfunction. The 10 point cutoff NOSE score, which is a quickly available tool without expensive medical cost, could determine which patients would most likely benefit from early application of intranasal treatment.

## Conflicts of interest

The authors declare no conflicts of interest.
